# Awareness of Current Surgical Practice for Congenital Scoliosis and Optimal Timing of Treatment Among Physicians and Patients’ Families in Saudi Arabia

**DOI:** 10.7759/cureus.68677

**Published:** 2024-09-04

**Authors:** Salma S Alshammasi, Alzahra B Almughlliq, Noor A Alqrunawi, Kawther A Alsheddi, Sultan Alsalmi

**Affiliations:** 1 Medicine and Surgery, College of Medicine, Imam Abdulrahman Bin Faisal University, Dammam, SAU; 2 Neurosurgery, Imam Abdulrahman Bin Faisal University, Dammam, SAU

**Keywords:** awareness, congenital scoliosis, optimal timing, physicians and patients’ families, surgical intervention

## Abstract

Background: Congenital scoliosis (CS) is a developmental spinal deformity characterized by an abnormal curvature of the spine, affecting one in 1,000 births. The mainstay of treatment involves either observation or surgery in significant curve progression. The optimal timing of surgical intervention is debated, with early intervention preferred. Therefore, understanding physicians' and patients’ families’ perspectives is crucial for optimizing surgical outcomes in CS.

Objective: To assess the awareness and knowledge of physicians and patients' families regarding current, as well as new surgical practices and the optimal timing of treatment for CS.

Methods: A cross-sectional study was conducted in Saudi Arabia using an online self-administered questionnaire distributed through social media platforms and neurosurgery clinics. Levels of awareness were assessed by a knowledge-scoring system.

Results: The study involved 403 participants, primarily patients' families (85.1%, N = 343) and physicians (14.9%, N = 60). The results show that physicians had a significantly higher correct response than patients' families regarding the ideal age for surgical correction of CS, the timing of surgical intervention whether before or after maturity, and the role of conservative management, as evident from statistically significant p-values of <0.001, 0.031, and <0.001, respectively. On the contrary, patients' families excelled in understanding interventions irrespective of symptomatic status if Cobb’s angle is 40 degrees or above, with a statistically significant p-value of 0.031. Both groups exhibited a good level of overall knowledge, as evidenced by mean awareness scores of 12.18 and 11.64, respectively. Additionally, physicians had a statistically significant higher level of awareness compared to patients’ families, with a p-value of (0.014). However, both groups demonstrated poor knowledge of the latest techniques, including distraction-based magnetically controlled growing rods (MCGRs), growth-guided modern Luque trolleys, and posterior dynamic deformity correction (ApiFix).

Conclusion: The mean awareness score of both physicians and patients' families indicates a good level of knowledge. However, both groups exhibited poor knowledge in relation to the optimal timing of treatment and new surgical techniques.

## Introduction

Congenital scoliosis (CS) is a complex spinal deformity that occurs during fetal development due to defects of formation and/or segmentation, resulting in abnormal curvature of the spine [1]. It is estimated that congenital scoliosis affects approximately one in 1,000 live births worldwide, making it a relatively rare condition [2].

The mainstay of treatment involves either observation or surgery in significant curve progression (>10°/year) [3]. However, it is important to note that not all scoliotic conditions necessitate bracing or surgical intervention. Approximately 25% of scolioses exhibit a low progression rate or compensated defects of formation, which typically do not require surgery and should be periodically evaluated [1]. On the other hand, around 75% of congenital scoliosis require surgical intervention, which is indicated between the ages of one and four years [1]. The decision to proceed with surgery is primarily based on the magnitude of the scoliotic curve, which is assessed using Cobb's angle. Patients with curves below 40° are carefully monitored every four to six months, while surgical intervention is essential for curves exceeding 40° [1].

The optimal timing of surgical treatment for congenital scoliosis remains a subject of debate among healthcare professionals. Generally, early intervention is preferred to guide spinal growth and prevent worsening of the deformity [2,3].

However, the timing of surgical treatment requires a collaborative effort between physicians and the families of affected individuals, with the awareness of current surgical practice playing a crucial role in making informed decisions about treatment options and timing [4]. By exploring the perspectives and understanding of both healthcare professionals and relatives of those directly impacted by the condition, we can identify potential gaps in knowledge and provide valuable insights regarding surgical practice for congenital scoliosis. This research aims to assess the level of awareness and knowledge regarding current surgical practices and optimal timing of treatment for congenital scoliosis among physicians and patients' families.

## Materials and methods

Study design

This is a cross-sectional study that was conducted in Saudi Arabia to assess the level of awareness and knowledge regarding current surgical practices and optimal timing of treatment for congenital scoliosis among physicians and patients' families. Data were collected using an online self-administered questionnaire sent via various social media platforms, including WhatsApp, Telegram, and X (Twitter previously). In addition, barcodes of the questionnaire were distributed among neurosurgery clinics in King Fahad Hospital of the University. The target population was physicians working in Saudi Arabia and families of patients with congenital scoliosis. Data collection took place over a period of four months, from December 2023 until the end of March 2024. A knowledge scoring system was used to assess the level of awareness for physicians and patients’ families. If fewer than 50% of the participants answered the question correctly, their knowledge is categorized as poor. If the question was answered correctly by 50-75% of participants, it is considered a fair level of knowledge. If more than 75% of participants answered the question correctly, it is considered good knowledge [5]. The same scoring system will be used to assess the mean score of awareness [5].

Study population

Inclusion criteria encompassed actively practicing Saudi physicians across diverse medical specialties and families of patients diagnosed with congenital scoliosis. Exclusion criteria included physicians not practicing in Saudi Arabia and families of patients diagnosed with idiopathic scoliosis, structural scoliosis, neuromuscular scoliosis, syndromic scoliosis, or any type of scoliosis other than congenital scoliosis. Convenience sampling was used, and the sample size of 383 was calculated using an online tool (raosoft.com), meeting the minimum requirement for a 95% confidence interval with a margin of error of ±5%.

Pilot study

A pilot study was conducted involving 38 participants, which represented 10% of the total sample, to evaluate the accuracy of the questionnaire. Participants in the pilot study were recruited randomly from a range of social media platforms such as WhatsApp, Telegram, and X (formerly Twitter), as well as from the neurosurgery cinics at King Fahad Hospital.

Ethical consideration

Ethical approval was obtained from the Institutional Review Board at Imam Abdulrahman bin Faisal University, Saudi Arabia (IRB#: IRB-UGS-2023-01-429).

Variables

Table 1 presents the illustration of the dependent and independent variables.

**Table 1:** Definition of variables.

Variable	Type	Scale of Measurement
Independent variable: to identify their relation to awareness of current surgical practice for congenital scoliosis and optimal timing of treatment among physicians and patients’ families		
Gender	Dichotomous	Male
		Female
Age	Categorical	15-29
		30-44
		45-59
		60 and above
Are you a physician?	Dichotomous	Yes
		No
Degree	Categorical	Non-educated
		High school
		Bachelor
		Master
		PhD
Region of residence	Categorical	Central region
		Eastern region
		Midwest region
		Northern region
		Northwest region
		Southwest region
Specialty	Categorical	Orthopedics
		Neurosurgery
		Other
Current title	Categorical	General practitioner
		Resident
		Specialist
		Senior registrar
		Consultant
Sector of practice	Categorical	Governmental sector
		Private sector
		Both
Years of experience	Categorical	1-5
		6-10
		11-15
		16 and above
Region of practice in Saudi Arabia	Categorical	Central region
		Eastern region
		Midwest region
		Northwest region
		Southwest region
Region of residence	Categorical	Central region
		Eastern region
		Midwest region
		Northern region
		Northwest region
		Southwest region
Dependent variables: to assess the awareness of current surgical practice for congenital scoliosis and optimal timing of treatment among physicians and patients’ families		
Self-administered questionnaire	Nominal	Percentage (%)

Materials

A questionnaire was structured to include the following sections:

Demographics: Gender, age, and participant status (physician or not). If a physician: specialty, current title, section of practice, years of experience, number of scoliosis operations per year, region of practice in Saudi Arabia, and region of residence. If not a physician: degree and region of residence.

Knowledge assessment: Optimal age of surgical correction for congenital scoliosis, the timing of surgical intervention (before or after maturity), the role of conservative management, replacement of surgical intervention by conservative treatment, the influence of curvature angle on intervention type, Cobb’s angle degree and indication for surgical intervention, the correlation between age increase and curvature progression, need for intervention regardless of symptoms, familiarity with correctional surgical techniques, preferred surgical fusion options, and factors contributing to delayed surgical intervention.

Pre-operative imaging: Options of pre-operative imaging.

Operative assessment: Optimal operative techniques for congenital scoliosis.

Post-operative assessment: Most common postoperative complications and correlation between age increase and incidence of postoperative complications.

Data analysis

Data were analyzed by using Statistical Product and Service Solutions (SPSS, version 22; IBM SPSS Statistics for Windows, Armonk, NY). Continuous variables were expressed as mean ± standard deviation and categorical variables were expressed as percentages. The Mann-Whitney test and Kruskal-Wallis test were used for continuous variables without normal distribution. The chi-square test was used for categorical variables. The Shapiro-Wilk test was used to assess normality distribution for the variables. A p-value <0.05 was considered statistically significant.

## Results

The study involved a total of 403 participants, consisting of physicians and patients' families. The majority of participants were patients' families, comprising 85.1% (N = 343), while physicians encompassed 14.9% (N = 60). Demographics showed that 71.2% (N =287) of participants were females, with 69.7% (N = 281) falling in the range of 15-29 years old. About half of the patients' families had attained a bachelor's degree or a higher education level. Regarding geographical distribution, 40.2% (N = 138) of patients' families reside in the Eastern Province. In terms of medical specialties, orthopedics and neurosurgery represented 23.3% (N = 14) of physicians, with consultants representing 35% (N = 21) of all physicians, the majority of whom worked in the governmental sector. Additionally, nearly two-thirds of physicians were based in the Eastern Province, with 48.3% (N = 29) of overall physicians (N = 60) reporting one to five years of experience (Table 2).

**Table 2:** Demographics and characteristics of physicians and patients’ families.

Demographics	Variable	N=403	% of participants
Gender	Male	116	28.8
	Female	287	71.2
Age	15-29	281	69.7
	30-44	84	20.8
	45-59	30	7.4
	60 and above	8	2.0
Are you a physician?	Yes	60	14.9
	No	343	85.1
Patients’ families		N=343	85.1
Degree	Non-educated	10	2.9
	High school	162	47.2
	Bachelor	160	46.6
	Master	10	2.9
	PhD	1	.3
Region of residence	Central region	63	18.4
	Eastern region	138	40.2
	Midwest region	33	9.6
	Northern region	21	6.1
	Northwest region	32	9.3
	Southwest region	56	16.3
Physicians		N=60	14.9
Specialty	Orthopedics	6	10.0
	Neurosurgery	8	13.3
	Other	46	76.7
Current title	General practitioner	11	18.3
	Resident	14	23.3
	Specialist	7	11.7
	Senior registrar	7	11.7
	Consultant	21	35.0
Sector of practice	Governmental sector	40	66.7
	Private sector	10	16.7
	Both	10	16.7
Years of experience	1-5	29	48.3
	6-10	8	13.3
	11-15	9	15.0
	16 and above	14	23.3
Region of practice in Saudi Arabia	Central region	8	13.3
	Eastern region	39	65.0
	Midwest region	7	11.7
	Northwest region	2	3.3
	Southwest region	4	6.7
Region of residence	Central region	8	13.6
	Eastern region	38	64.4
	Midwest region	7	11.9
	Northern region	1	1.7
	Northwest region	1	1.7
	Southwest region	4	6.8

**Figure 1:**
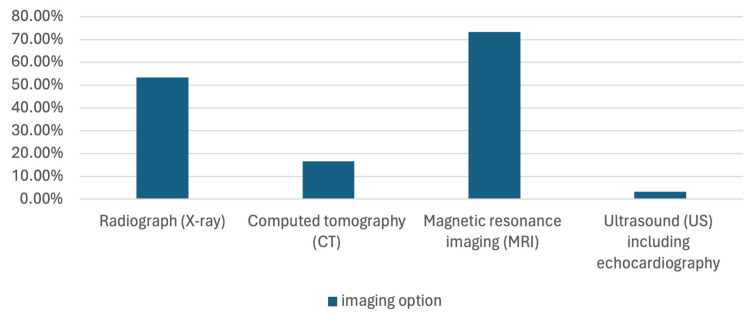
This graph shows the responses of physicians to what are the options for pre-operative imaging.

In the assessment of physicians' knowledge regarding the surgical management of CS, it was found that physicians had good knowledge regarding the role of conservative management and how it does not replace surgical management. Additionally, physicians who participated had a good knowledge of how the angle of curvature influences the type of intervention; however, they had poor knowledge in regard to the specific degree of curvature that intervention is considered. Furthermore, it was found that 85% (N = 51) of participants exhibited poor knowledge in relation to the optimal age for surgical correction, while 68.33% (N = 41) demonstrated fair knowledge regarding the timing of surgical intervention in relation to maturity. The majority of physicians 96.67% (N = 58) had good knowledge about the correlation between aging and spinal curvature progression. Moreover, only 46.67% (N = 28) of physicians were aware that surgical intervention should be performed regardless of whether the patient is symptomatic or not; therefore, they are considered to have poor knowledge. Physicians were mostly familiar with in situ fusion and hemiepiphysiodesis, followed by hemivertebra resection as surgical techniques used for the correction of CS. However, they showed poor knowledge in relation to the familiarity with the newest techniques, which are distraction-based magnetically controlled growing rods (MCGRs), growth-guided modern Luque trolleys, posterior dynamic deformity correction (ApiFix), and compression-based vertebral body stapling, and compression-based vertebral body tethering. Physicians had good knowledge concerning the best fusion option and the majority believe that delayed surgical intervention occurs due to the fact that early intervention affects normal skeletal growth. Physicians believe that the optimal operative technique is in situ fusion and hemiepiphysiodesis. For the physicians’ postoperative assessment, most of the physicians believe that wound complications are the most common complications of the surgical management of CS. Most physicians reported that complications increase as age increases, which indicates good knowledge (Table 3). In relation to the preoperative assessment, physicians were mostly familiar with MRI as an imaging technique done preoperatively, followed by X-ray and CT scans (Figure 1).

**Table 3:** Responses of the physicians regarding current surgical practice for congenital scoliosis and optimal timing of treatment.

Questions for physicians regarding current surgical practice for congenital scoliosis and optimal timing of treatment	Options	N= 60	% of participants
When do you think is the optimal age for surgical correction for congenital scoliosis?	1 - 4years	9	15.00
	> 4 years	51	85.00
Do you think it is better to surgically intervene before maturity or after it?	Before maturity	41	68.33
	After maturity	19	31.67
Do you think that conservative management has a role in treating congenital scoliosis?	Yes	52	86.67
	No	8	13.33
Does conservative treatment replace the need for surgical intervention?	Yes	14	23.33
	No	46	76.67
Do you think that the angle of curvature influences the type of intervention?	Yes	59	98.33
	No	1	1.67
At what Cobb’s angle a surgical intervention should be considered?	10-20 degrees	3	5.00
	20-40 degrees	28	46.67
	More than 40 degrees	29	48.33
Is there a correlation between an increase in age and the progression of spinal curvature?	Yes	58	96.67
	No	2	3.33
Should interventions be performed regardless of whether the patient is symptomatic or not?	Yes	28	46.67
	No	32	53.33
What types of congenital scoliosis correctional techniques you are familiar with?			
In situ fusion and hemiepiphysiodesis		30	50.00
Hemivertebra resection		26	43.33
Distraction-based vertical		23	38.33
Expandable prosthetic titanium		1	1.67
Distraction-based traditional/conventional growing rods		24	40.00
Distraction-based magnetically controlled growing rods		11	18.33
Compression-based vertebral body stapling		8	13.33
Compression-based vertebral body tethering		9	15.00
Growth-guided Shilla system		8	13.33
Growth-guided modern Luque trolley		4	6.67
Posterior dynamic deformity correction (ApiFix)		6	10.00
What do you think is the better surgical fusion option for congenital scoliosis?	Long segment fusion	23	38.33
	Short segment fusion	37	61.67
What factors do you believe contribute to the delayed surgical intervention for congenital scoliosis?			
Because early intervention affects normal skeletal growth		19	31.67
Thinking that conservative intervention is better		8	13.33
Complication rate increases with early surgical intervention		6	10.00
Lack of knowledge of optimal treatment time		11	18.33
Families’ fear of surgical intervention		7	11.67
Availability of congenital scoliosis screening		5	8.33
Far scheduled appointments		1	1.67
No access to medical facilities		1	1.67
What are the options for pre-operative imaging?			
Radiograph (X-ray)		32	53.33
Computed tomography (CT)		10	16.67
Magnetic resonance imaging (MRI)		44	73.33
Ultrasound (US) including echocardiography		2	3.33
What are the optimal operative techniques to be performed for congenital scoliosis?			
In situ fusion and hemiepiphysiodesis		26	43.33
Hemivertebra resection		8	13.33
Distraction-based vertical		10	16.67
Compression-based vertebral body stapling		2	3.33
Compression-based vertebral body tethering		1	1.67
Posterior dynamic deformity correction		4	6.67
What are the most common post-operative complications after congenital scoliosis surgery?			
Wound complications		33	55.00
Implant related issues		23	38.33
Neurological complications		30	50.00
Respiratory problems		7	11.67
Pseudoarthrosis		2	3.33
Crankshaft phenomenon		4	6.67
Is there a correlation between the increase in age and a higher incidence of post-operative complications?	Yes	49	81.67
	No	11	18.33

**Figure 2:**
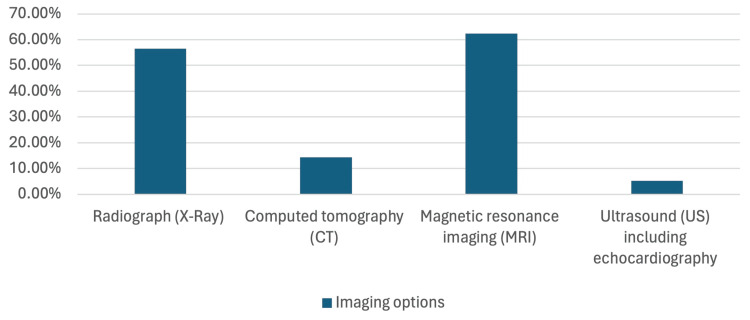
This figure shows the responses of patients’ families of what are the options of preoperative options.

In the assessment of patients’ families’ knowledge regarding the surgical management of CS, it was found that almost all families (97.96%, N= 336) were poorly knowledgeable of the optimal timing for the surgical correction of CS. On the contrary, families had a fair knowledge of surgical intervention before maturity, the role of conservative management, and the irreplaceability of surgical management. In addition, a great number of families who participated had a good knowledge in relation to the influence of angle’s curvature on the type of intervention and on the correlation between the increase in age and progression of spinal curvature, with percentages of 92.13% (N = 316) and 91.25% (N = 313), respectively. Families possessed a fair knowledge about Cobb’s angle that requires surgical intervention and the requirement of surgical intervention whether the patient is symptomatic or not. They also had a fair knowledge of short-segment fusion being the better surgical option. Families were mostly familiar with distraction-based: vertical expandable prosthetic titanium rib, followed by in situ fusion and hemiepiphysiodesis as surgical techniques used for the correction of CS. However, they showed poor knowledge in relation to the familiarity with the newest techniques, which are distraction-based MCGRs, growth-guided modern Luque trolleys, ApiFix, and compression-based vertebral body stapling, and compression-based vertebral body tethering. Families believe that the optimal operative technique is in situ fusion and hemiepiphysiodesis. Families believe that factors that contributed to the delay of surgical intervention were families’ fear of surgical intervention, followed by the fact that early intervention affects normal skeletal growth and the lack of availability of CS screening. The most common postoperative complications that were known after CS surgery were neurological complications, followed by wound complications. On the other hand, families had a fair knowledge of the fact that an increase in age is associated with a higher incidence of post-operative complications (Table 4). In relation to the preoperative assessment, families were predominantly knowledgeable about MRI as a diagnostic imaging modality conducted prior to surgery, followed by X-ray and CT scans (Figure 2).

**Table 4:** Responses of the patients’ families regarding current surgical practice for congenital scoliosis and optimal timing of treatment.

Questions for the patients’ families regarding current surgical practice for congenital scoliosis and optimal timing of treatment.	Options	N=343	% of participants
When do you think is the optimal age for surgical correction for congenital scoliosis?	1 - 4years	7	2.04
	> 4 years	336	97.96
Do you think it is better to surgically intervene before maturity or after it?	Before maturity	183	53.35
	After maturity	160	46.65
Do you think that conservative management has a role in treating congenital scoliosis?	Yes	194	56.56
	No	149	43.44
Does conservative treatment replace the need for surgical intervention?	Yes	92	26.82
	No	251	73.18
Do you think that the angle of curvature influences the type of intervention?	Yes	316	92.13
	No	27	7.87
At what Cobb’s angle a surgical intervention should be considered?	10-20 degree	27	7.87
	20-40 degree	112	32.65
	More than 40 degree	204	59.48
Is there a correlation between the increase in age and the progression of spinal curvature?	Yes	313	91.25
	No	30	8.75
Should interventions be performed regardless of whether the patient is symptomatic or not?	Yes	211	61.52
	No	132	38.48
What types of congenital scoliosis correctional techniques you are familiar with?			
In situ fusion and hemiepiphysiodesis		109	31.78
Hemivertebra resection		73	21.28
Distraction-based vertical		142	41.40
Expandable prosthetic titanium		17	4.96
Distraction-based traditional/conventional growing rods		77	22.45
Distraction-based magnetically controlled growing rods		49	14.29
Compression-based vertebral body stapling		69	20.12
Compression-based vertebral body tethering		40	11.66
Growth-guided Shilla system		12	3.50
Growth-guided modern Luque trolley		14	4.08
Posterior dynamic deformity correction (ApiFix)		26	7.58
What do you think is the better surgical fusion option for congenital scoliosis?	Long segment fusion	162	47.23
	Short segment fusion	181	52.77
What factors do you believe contribute to the delayed surgical intervention for congenital scoliosis?			
Because early intervention affects normal skeletal growth		56	16.33
Thinking that conservative intervention is better		46	13.41
Complication rate increases with early surgical intervention		25	7.29
Lack of knowledge of optimal treatment time		32	9.33
Physician’s recommendation to delay the surgical intervention		16	4.66
Families’ fear of surgical intervention		72	20.99
Availability of congenital scoliosis screening		48	13.99
Far scheduled appointments		30	8.75
No access to medical facilities		14	4.08
What are the options for pre-operative imaging?			
Radiograph (X-ray)		194	56.56
Computed tomography (CT)		49	14.29
Magnetic resonance imaging (MRI)		214	62.39
Ultrasound (US) including echocardiography		18	5.25
What are the optimal operative techniques to be performed for congenital scoliosis?			
In situ fusion and hemiepiphysiodesis		121	35.28
Hemivertebra resection		23	6.71
Distraction-based vertical		79	23.03
Compression-based vertebral body stapling		27	7.87
Compression-based vertebral body tethering		16	4.66
Posterior dynamic deformity correction		26	7.58
What are the most common post-operative complications after congenital scoliosis surgery?			
Wound complications		152	44.31
Implant-related issues		84	24.49
Neurological complications		157	45.77
Respiratory problems		64	18.66
Pseudoarthrosis		12	3.50
Crankshaft phenomenon		58	16.91
Is there a correlation between the increase in age and a higher incidence of post-operative complications?	Yes	255	74.34
	No	88	25.66

The findings indicate that physicians demonstrated a notably higher percentage of correct responses compared to patients’ families in regard to questions surrounding optimal age for surgical correction of CS, timing of surgical intervention before or after maturity, and the role of conservative management, supported by the statistical significance of p-values of <0.001, 0.031, and <0.001, respectively. Conversely, patients' families exhibited a significantly higher correct response rate regarding interventions regardless of symptomatic status, with a statistically significant p-value of 0.031 (Table 5).

**Table 5:** Percentage of the correct answers for the questionnaire of awareness of current surgical practice for congenital scoliosis and optimal timing of treatment among physicians and patients’ families.

	All (N=403)				Physicians (N=60)				Patients’ families (N=343)				P value
	Correct		Not correct		Correct		Not correct		Correct		Not correct		
	Number	%	Number	%	Number	%	Number	%	Number	%	Number	%	
When do you think is the optimal age of surgical correction for congenital scoliosis?	16	3.97	387	96.03	9	15.00	51	85.00	7	2.04	336	97.96	<0.001*
Do you think it is better to surgically intervene before maturity or after it?	224	55.58	179	44.42	41	68.33	19	31.67	183	53.35	160	46.65	0.031*
Do you think that conservative management has a role in treating congenital scoliosis?	246	61.04	157	38.96	52	86.67	8	13.33	194	56.56	149	43.44	<0.001*
Does conservative treatment replace the need for surgical intervention?	297	73.70	106	26.30	46	76.67	14	23.33	251	73.18	92	26.82	0.571
Do you think that the angle of curvature influences the type of intervention?	375	93.05	28	6.95	59	98.33	1	1.67	316	92.13	27	7.87	0.081
At what Cobb’s angle a surgical intervention should be considered?	233	57.82	170	42.18	29	48.33	31	51.67	204	59.48	139	40.52	0.107
Is there a correlation between increase in age and progression of spinal curvature?	371	92.06	32	7.94	58	96.67	2	3.33	313	91.25	30	8.75	0.153
Should interventions be performed regardless of whether the patient is symptomatic or not?	239	59.31	164	40.69	28	46.67	32	53.33	211	61.52	132	38.48	0.031*
What types of congenital scoliosis correctional techniques you are familiar with?	403	100.00	0	0.00	60	100.00	0	0.00	343	100.00	0	0.00	-
What do you think is the better surgical fusion option for congenital scoliosis?	403	100.00	0	0.00	60	100.00	0	0.00	343	100.00	0	0.00	-
What factors do you believe contribute to the delayed surgical intervention for congenital scoliosis?	403	100.00	0	0.00	60	100.00	0	0.00	343	100.00	0	0.00	-
What are the options for pre-operative imaging?	403	100.00	0	0.00	60	100.00	0	0.00	343	100.00	0	0.00	-
What are the optimal operative techniques to be performed for congenital scoliosis?	403	100.00	0	0.00	60	100.00	0	0.00	343	100.00	0	0.00	-
What are the most common post-operative complications after congenital scoliosis surgery?	403	100.00	0	0.00	60	100.00	0	0.00	343	100.00	0	0.00	-
Is there a correlation between increase in age and higher incidence of post-operative complications?	304	75.43	99	24.57	49	81.67	11	18.33	255	74.34	88	25.66	0.224

The results indicate that the mean score of awareness regarding current surgical practices for CS and the optimal timing of treatment differs among physicians and patients' families based on their characteristics. If the mean score awareness score is below 7.5, the population's overall knowledge is considered poor. If the score falls between 7.5 and 11.25, it is considered as a fair knowledge. Scores exceeding 11.25 indicate a good level of knowledge. Notably, while there were no statistically significant differences observed in the mean scores across most participant characteristics, gender emerged as a key differentiating factor with a statistically significant p-value of (p=0.009). Moreover, physicians demonstrated a higher awareness score compared to patients' families with a statistically significant p-value of (p=0.014). Both physicians and patients' families demonstrated a good level of overall knowledge, with mean awareness scores of 12.18 and 11.64, respectively (Table 6).

**Table 6:** Mean score of awareness of current surgical practice for congenital scoliosis and optimal timing of treatment among physicians and patients’ families by their characteristics.

Variables		Mean overall score (out of 15)	SD	P value
Mean score of all participants		11.72	1.28	-
Gender	Male	12.01	1.31	0.009*
	Female	11.60	1.25	
Age	15-29	11.71	1.31	0.528
	30-44	11.83	1.21	
	45-59	11.47	1.28	
	60 and above	11.88	0.64	
Are you a physician?	Yes	12.18	1.28	0.014*
	No	11.64	1.26	
Patients’ families				
Degree	Non-educated	10.90	2.28	0.313
	High school	11.56	1.29	
	Bachelor	11.80	1.11	
	Master	11.10	1.52	
	PhD	12.00	-	
Region of residence	Central region	11.83	1.21	0.447
	Eastern region	11.52	1.29	
	Midwest region	11.61	1.39	
	Northern region	11.10	1.84	
	Northwest region	11.75	1.05	
	Southwest region	11.88	0.95	
Physicians				
Specialty	Orthopedics	12.33	1.86	0.947
	Neurosurgery	12.13	1.25	
	Other	12.17	1.23	
Current title	General practitioner	12.36	1.29	
	Resident	12.07	1.38	0.921
	Specialist	12.57	1.81	
	Senior registrar	11.86	0.90	
	Consultant	12.14	1.20	
Sector of practice	Governmental sector	12.23	1.29	0.219
	Private sector	11.60	1.17	
	Both	12.60	1.26	
Years of experience	1-5	12.24	1.38	0.890
	6-10	11.88	1.46	
	11-15	12.44	1.33	
	16 and above	12.07	1.00	

## Discussion

In recent years, there have been numerous advancements in the surgical treatment of scoliosis, with the introduction of new surgical technologies, utilization of bone substitutes, monitoring of spinal cord function, and implementation of blood conservation strategies [6]. All these factors contribute to the complexity surrounding the understanding of surgeons and families of patients regarding optimal surgical management [7]. This study investigated the variations in awareness of the surgical management and optimal timing of treatment of congenital scoliosis among physicians and families of affected individuals. The study investigated the knowledge of physicians and patients' families regarding the surgical management of CS. Results revealed varying levels of knowledge among participants. Physicians showed good awareness of certain aspects such as the role of conservative management and the influence of the curvature’s angle on intervention types. However, they demonstrated poor knowledge regarding specific degrees of curvature warranting intervention, the optimal age for surgical correction, and the newest surgical techniques [1,8]. On the other hand, patients' families exhibited fair to good knowledge on several aspects, namely, the influence of age on spinal curvature progression and the necessity of surgical intervention regardless of symptoms if Cobb’s angle is 40° or above [1]. Additionally, both groups showed poor knowledge regarding the newest surgical techniques [8]. Notably, while physicians showed higher awareness overall, families were more aware of the need for intervention regardless of symptomatic status.

One of our hypotheses suggests that physicians and families of affected patients have a low level of awareness regarding current surgical practice for congenital scoliosis and the optimal timing of treatment. However, our study findings have led us to reject this hypothesis, as both physicians and patients' families demonstrated a good level of knowledge, with mean awareness scores of 12.18 and 11.64, respectively. On the other hand, the level of knowledge regarding new surgical techniques was found to be poor in both groups. The other hypothesis suggests that physicians would have greater knowledge overall compared to patients’ families, given their medical training and personal experiences. Our study findings have led us to accept this hypothesis as it was illustrated that the physicians have a statistically significant higher mean score of awareness than patients’ families (p-value = 0.014).

The findings of this study shed light on the beliefs held by patients' families that contribute to delayed surgical intervention for CS. It is crucial to consider these beliefs and their impact on treatment decisions in order to provide comprehensive care for individuals with CS. One significant belief identified among patients' families is the fear of surgical intervention. This fear may arise from concerns about the potential risks and complications associated with surgical intervention. Another belief highlighted in this study is the perception that early intervention may have negative effects on normal skeletal growth. This belief suggests a concern regarding their child's development. Additionally, the belief in the superiority of conservative interventions over surgical interventions for their children is another factor. Patients' families may have a preference for non-surgical approaches due to concerns about the invasiveness and potential complications associated with surgery. Moreover, a lack of knowledge of the optimal timing of treatment can lead to a delay in seeking healthcare providers and, therefore, delayed intervention which results in complex surgeries [1], progression of the complexity of the curve, diminished optimal outcomes, and increased post-operative complications [9]. Furthermore, delayed surgery can lead to severe local deformities and secondary structural curves [10]. The optimal time for surgery is a matter of debate. However, the literature reports well-established results in patients with CS who underwent surgery before the age of 10 [10]. On the contrary, in Sauri-Barraza's [11] study, it has been stated that multiple complications are attributed to early surgical intervention, and it was advised to postpone fusion surgery until maturity [11]. Addressing patients' families' concerns and providing accurate information about the safety and efficacy of surgical interventions can help alleviate concerns and promote timely intervention when necessary [4]. Furthermore, educating patients' families about the limitations of conservative interventions and the potential benefits of surgical intervention [12].

Additionally, recognizing the new surgical techniques is one of the main measures of this study. Physicians and patients' families showed poor knowledge of the newest surgical techniques used for CS. The insufficient knowledge regarding these surgical techniques in both physicians and patients' families indicates that these surgical techniques are not implemented in current surgical practice of CS in Saudi Arabia. Hence, it is essential to increase awareness regarding the latest advancements in surgical procedures to ensure that patients receive the best possible care and outcomes. In Bednar et al.'s [13] systemic review and meta-analysis comparing the newest magnetically controlled growing rods (MCGRs) with other distraction-based surgical technologies, it yielded that MCGRs had substantially lower complication rates and were as clinically effective as other technologies [13]. On the contrary, Lebel et al.'s [14] study results showed good deformity control with a high complication rate in MCGRs [14]. There is a lack of research regarding other recent techniques. However, it is worth mentioning that, in a published study by Froehlich et al. [15] about surgical treatment of adolescent idiopathic scoliosis with ApiFix, a 24-month follow-up result showed high improvement in the postoperative angles of the curvature [15]. Larson's [16] study emphasized the essential knowledge that primary care providers should possess [16]. It was stated that there is clearly a lack of proper screening for scoliosis among primary healthcare physicians [16]. This in turn could lead to a delay in the referral and consultation to neurosurgery for CS cases. In addition, the delay in referral could be attributed to the lack of knowledge regarding the optimal timing of surgical intervention.

This study contributes to the existing literature by providing valuable insights into the awareness of current surgical practice and optimal timing of treatment for congenital scoliosis among physicians and patients' families. To the best of our knowledge, there are no previous articles that specifically address this topic and examine the knowledge levels of both healthcare professionals and patients' families in this context. By filling this gap in the literature, our study enhances understanding of the current state of awareness and knowledge in this field. The findings can serve as a foundation for future research and the development of educational interventions aimed at improving awareness and promoting evidence-based decision-making in the surgical management of congenital scoliosis.

Limitations

The sample size of physicians and surgeons is limited compared to the sample of congenital scoliosis patient’s families. A greater number of neurosurgeons and orthopedic surgeons could have been a great aid to assess the current surgical practice used for congenital scoliosis and to properly compare the awareness with patients’ families.

Recommendations

It is advised to conduct additional research with a specific emphasis on the degree of curvature and the optimal techniques employed. More research should be conducted due to the lack of knowledge of new surgical practices for managing congenital scoliosis in Saudi Arabia. There is no international standardization among professional spinal surgeons regarding the ideal surgical method and optimal age for treating congenital scoliosis. Therefore, more studies will help improve the quality of care for individuals with this condition, improving their outcomes and quality of life.

## Conclusions

Congenital scoliosis is a lateral curvature of the spine arising from vertebral anomalies that are present at birth. Identification of this condition from an early age is a cornerstone for the management. However, the majority of physicians and patients’ families demonstrated a poor knowledge level of the optimal timing to perform a surgical intervention for congenital scoliosis. They also demonstrated a poor level of knowledge in relation to familiarity with the newest techniques, which are distraction-based MCGRs, growth-guided modern Luque trolleys, ApiFix, compression-based vertebral body stapling, and compression-based vertebral body tethering. In conclusion, an early intervention could potentially impact the outcome and complications of the surgery. It is also crucial to develop a better understanding of the optimal timing for surgical intervention of CS.
